# Activating cGAS-STING pathway for the optimal effect of cancer immunotherapy

**DOI:** 10.1186/s13045-019-0721-x

**Published:** 2019-04-01

**Authors:** Anping Li, Ming Yi, Shuang Qin, Yongping Song, Qian Chu, Kongming Wu

**Affiliations:** 1 Department of Oncology, The First Affiliated Hospital of Zhengzhou University, Zhengzhou, 450052 China; 2 Department of Oncology, Tongji Hospital of Tongji Medical College, Huazhong University of Science and Technology, Wuhan, 430030 China; 3 Department of Hematology, the Affiliated Cancer Hospital of Zhengzhou University, Henan Cancer Hospital, Zhengzhou, 450000 Henan China

**Keywords:** cGAS-STING, Innate immunity, Type I interferon, Cancer immunotherapy, CAR-T, Immune checkpoint inhibitor

## Abstract

During tumor progression, a subset of cancer cells escape from immune surveillance and eventually develop into measurable tumor mass. Cancer immunotherapy eradicates tumor cells by enhancing multiple steps in cancer-immunity cycle including antigen presentation, T cell priming, activation, and immune killing activity. Immunotherapy has been verified as an effective strategy in multiple cancers, but some problems still exist in actual clinical practice such as frequent primary and adaptive resistance. Combination with other adjuvant therapies gives us a new perspective to overcome the emerging obstacles in immunotherapy application. Recently, a series of studies demonstrated that the vital component of host innate immunity — cGAS-STING pathway might play an important role in anti-cancer immunity. It is generally acknowledged that the downstream signals of cGAS-STING especially type I interferon (IFN) bridge innate immunity and adaptive immunity. Given the functions of type I IFN in promoting the maturation and migration of dendritic cells, enhancing cytotoxic T lymphocyte- or natural killer cell-mediated cytotoxicity effect, and protecting effector cells from apoptosis, we believe cGAS-STING agonist might be used as sensitizer for multiple immunotherapies such as cancer vaccine, immune checkpoint blockade, and chimeric antigen receptor T cell therapy. In this review, we highlight the latest understanding of cGAS-STING pathway and the advances of the combination therapy of STING agonist and immunotherapy.

## Introduction

Accumulating mutations in cancer cells not only render malignant transformation, but also activate host’s anti-tumor immune response. However, under selective pressure, cancer cells with high immunogenicity are eliminated while ones with low immunogenicity survive. This process is called immunoediting [1]. Dynamically evolving antigen spectrum endows cancer with the capability of immune escape [2]. Apart from immunoediting, other factors such as immunosuppressive tumor microenvironment contribute to immune evasion as well [3]. Cancer immunotherapy is developed to counteract multiple inhibitory immune factors, from impaired cancer antigen presentation to unleashed cancer-killing activity [4–6]. During past few decades, multiple cancer immunotherapies have been successfully applied in clinical practice including oncolytic virus, chimeric antigen receptor T cell (CAR-T), bispecific antibody, and immune checkpoint inhibitor (ICI) [7–10]. Most immunotherapies are aiming to enhance adaptive anti-tumor immunity.

Actually, adaptive anti-tumor immunity is highly dependent on robust innate immunity [11]. As the first immune barrier of host, innate immunity could sense non-self-material by various pattern recognition receptors (PRRs) such as cytosolic DNA sensor [12]. Malignant transformation usually accompanies formation of cytosolic chromatin fragments and micronuclei, increasing the probability of DNA leakage in cancer cell or cancer cell-derived DNA uptake by dendritic cell (DC) [13]. Stimulated by cytosolic DNA, active cyclic GMP-AMP synthase-stimulator of interferon genes (cGAS-STING) pathway stimulates the expression of type I interferon (IFN) in cancer cells or DCs, initiating innate anti-cancer immunity [13–16]. Actually, type I IFN is a versatile molecule related with cell senescence and inflammation response [17]. It has been verified that type I IFN signal is essential to the cross-priming of the tumor-specific T cells [18].

Since STING molecule was found in 2008 [19, 20], substantial efforts have been expended to find an appropriate cGAS-STING agonist for anti-cancer agent development. Actually, cGAS-STING agonists not only induce cancer cell senescence but enhance adaptive anti-cancer immunity which would synergize with immunotherapies [21–24]. In this review, we highlight the latest understanding and the advances of cGAS-STING-targeting strategies, especially in combination with immunotherapies such as cancer vaccine, ICI, oncolytic virus, and chimeric antigen receptor T cell (CAR-T) therapy.

### The role of cGAS-STING pathway in anti-cancer immunity

#### cGAS-STING pathway

**Fig. 1:**
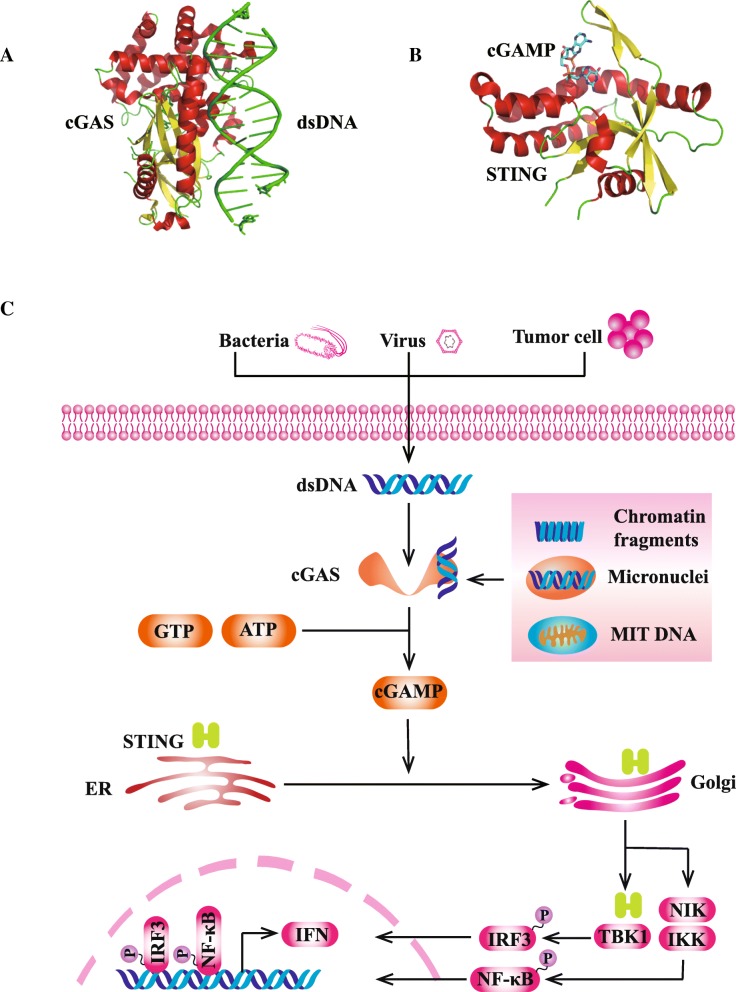
cGAS-STING pathway. **a** Three-dimensional structure of cGAS-dsDNA complex. **b** Three-dimensional structure of STING. The above structures were presented by PyMOL Molecular Graphics System. **c** cGAS-STING pathway. Cytosolic DNA sensor could be activated by exogenous DNA derived from bacteria, virus, or damaged/dying tumor cell. In addition, cGAS could sense endogenous chromosomal fragments, as well as DNA leakage from micronuclei and mitochondria. cGAS directly binds to tumor-derived dsDNA and subsequently catalyzes the production of cyclic GMP-AMP (cGAMP). Following the stimulation of cGAMP, the conformation of STING molecule is changed from monomer to dimers. Then, STING dimers are translocated to perinuclear microsome from ER via Golgi apparatus. STING could recruit and activate TANK-binding kinase 1 (TBK1) which further phosphorylates interferon regulatory transcription factor 3 (IRF3) and upregulates the expression of type I IFN. In addition, STING could activate nuclear factor kappa-light-chain-enhancer of activated B cells (NF-κB) pathway by binding to IκB kinase (IKK) and NF-κB-inducing kinase (NIK). Activated NF-κB pathway collaborates with TBK1-IRF3 pathway to induce the expression of type I IFN. Abbreviations: cGAMP, cyclic GMP-AMP; ER, endoplasmic reticulum; IKK, IκB kinase; IRF3, interferon regulatory transcription factor 3; MIT, mitochondria; NF-κB, nuclear factor kappa-light-chain-enhancer of activated B cells; NIK, NF-κB-inducing kinase; TBK1, TANK-binding kinase 1

STING is a cytosolic DNA sensor anchored in endoplasmic reticulum (ER) [25–27]. STING pathway could not be directly activated by double-stranded DNA (dsDNA). Instead, STING pathway is predominantly activated by second messenger cyclic dinucleotide (CDN) which is generated by cGAS [28]. Cytosolic dsDNA directly binds to cGAS and subsequently catalyzes the production of cyclic GMP-AMP (cGAMP) (Fig. 1a) [29, 30]. Following the stimulation of cGAMP, the conformation of STING molecule is changed from monomer to dimers (Fig. 1b) [13]. Then, STING dimers are translocated to perinuclear microsome from ER via Golgi apparatus [13]. STING could recruit and activate TANK-binding kinase 1 (TBK1) which further phosphorylates interferon regulatory transcription factor 3 (IRF3) and upregulates the expression of type I IFN [31]. In addition, STING could activate nuclear factor kappa-light-chain-enhancer of activated B cells (NF-κB) pathway by binding to IκB kinase (IKK) and NF-κB-inducing kinase (NIK) [32, 33]. Activated NF-κB pathway collaborates with TBK1-IRF3 pathway to induce the expression of type I IFN (Fig. 1c) [13]. Type I IFN has multiple immune-stimulatory functions promoting the maturation, migration, and activation of multiple immune cells such as DC, T cell, and natural killer cell (NK) [18].

Apart from recognizing foreign DNA, cGAS-STING pathway could sense self-DNA derived from damaged and dying cells, which contributes to sterile inflammation in the context of autoimmune diseases and anti-cancer immunity [18]. It is generally believed that cancer-derived DNA could get in DC cytoplasm and stimulate cGAS-STING-type I IFN pathway, which is essential to subsequent activation of T cell [18]. Notably, the biological effect of cGAMP-STING pathway is not limited in a single cell. Both extracellular type I IFN and cell-cell transferred cGAMP could induce regional immune response [34].

As mentioned above, cGAS surveillance occurs in cytoplasm. However, the function of cGAS changes along with its subcellular location [35]. Distinguished from cytoplasmic cGAS, nuclear cGAS could interfere with the formation of PARP-Timeless complex and impede homologous recombination [35]. Nuclear shuttle of cGAS is induced by DNA damage, eventually leading to genome instability and malignant transformation [35]. Therefore, intracellular regulators of cGAS translocation such as B-lymphoid tyrosine kinase and karyopherin2 would be predictive biomarkers and treatment targets for a subset of cancer patients [35].

#### cGAS-STING pathway in cancer cell

For normal eukaryotic cell, DNA is strictly separated from cytoplasm to avoid auto-inflammation [36]. However, in tumor cell, the probability of exposure of DNA to cytosolic DNA sensor increases [37–39]. Even though the mechanisms by which nuclear DNA leaks into cytoplasm have not been completely understood, some factors are speculated to contribute to initiate endogenous DNA sensing [40]. Among these factors, the frequent formation of micronuclei plays a vital role in cGAS surveillance [40]. Due to the characteristic of genome instability, cancer cells usually undergo chromosome mis-segregation during cell division [41, 42]. The lagging chromosomes are enveloped by original nuclear membrane which further forms micronuclei [40, 43]. Micronuclei membrane is easy to break down and results in the release of contained dsDNA [44]. Besides micronuclei, small DNA fragments derived from DNA damage could be released into cytoplasm during the whole interphase which could activate cGAS-STING pathway as well [45–48]. In the context of reactive oxygen species (ROS) response, mitochondrial DNA leakage is another contributing factor to stimulating cytosolic DNA sensor and activating STING signaling [49].

The results of co-culture of cancer cells and effector cells showed that cancer cells with downregulated cGAS-STING pathway could resist to immune killing [50]. Further study in mouse model demonstrated that downregulated cGAS-STING pathway led to decreased tumor-infiltrating CD3^+^ CD8^+^ T cells by reducing the expression of downstream genes of type I IFN such as chemokine (C-X-C motif) ligands 9 and 10 (*CXCL9* and *CXCL10*) [50]. Independent of enhanced anti-cancer immunity, cGAS-STING pathway could directly activate senescence and apoptosis signaling pathways in cancer cells [51, 52]. cGAS-STING pathway downregulates the expression of anti-apoptosis protein BCL2 and upregulates the abundance of pro-apoptosis protein BCL2-associated X (BAX) [52]. BAX-mediated mitochondrial outer membrane permeabilization and simultaneous caspase-9-driven caspase-3 activation contribute to cell apoptosis [53]. Therefore, intact cGAS-STING pathway is an important regulator of cancer cell growth, senescence, and immune surveillance. As the consequence of selective pressure, surviving cancer cells tend to harbor deficiencies in cGAS-STING pathway. It has been detected that the activation of cGAS-STING is usually impaired in multiple cancers by epigenetic hypermethylation [46].

#### cGAS-STING pathway in DC

**Fig. 2:**
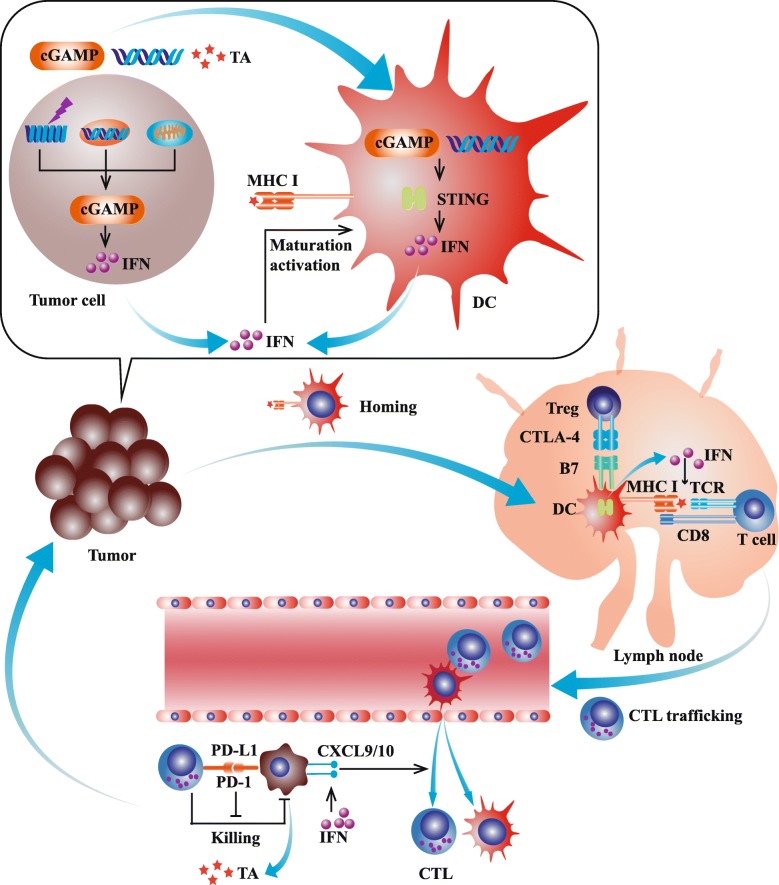
The role of cGAS-STING pathway in anti-tumor immunity. The cGAS-STING pathway upregulates multiples steps in cancer-immunity cycle. DNA leakage not only activates STING pathway in tumor cell, but also promotes STING activation in dendritic cell by DNA uptake or cGAMP transfer. In tumor microenvironment, cGAS-STING in DC plays an important role in the cross-presentation and priming of tumor-specific CD8^+^ T cell. Tumor-derived DNA could be taken up by DC like protein antigen, resulting in the following upregulation of type I IFN. Type I IFN contributes to most biological effects of cGAS-STING pathway on immune cells. Firstly, type I IFN reinforces the cross-presentation of DC by promoting antigen retention and CD8α+ DC survival. Besides, DC cultured with type I IFN shows increased expression of CCR7 which indicates improved lymph node-homing capability. In addition, type I IFN upregulates the expression of multiple Th1 chemokines including CXCL9 and CXCL10 which is important for the homing of APC and trafficking of cytotoxic T lymphocytes. Abbreviations: cGAMP, cytosolic GMP-AMP; CTL, cytotoxic T lymphocytes; CTLA-4, cytotoxic T-lymphocyte-associated protein 4; CXCL9, chemokine (C-X-C motif) ligand 9; IFN, interferon; MHC, major histocompatibility complex; PD-1/PD-L1, anti-programmed death-1/programmed death-ligand 1; TA, tumor antigen; TCR, T cell receptor; Treg, regulatory T cell

In tumor microenvironment, cGAS-STING in DC plays an important role in the cross-presentation and priming of tumor-specific CD8^+^ T cell (Fig. 2). Tumor-derived DNA could be taken up by DC like protein antigen, resulting in the following upregulation of type I IFN [54]. Type I IFN contributes to most biological effects of cGAS-STING pathway on immune cells. Firstly, type I IFN reinforces the cross-presentation of DC by promoting antigen retention and CD8α^+^ DC survival [35]. Besides, DC cultured with type I IFN shows increased expression of CCR7 which indicates improved lymph node-homing capability [55]. In addition, type I IFN upregulates the expression of multiple Th1 chemokines including CXCL9 and CXCL10 which is important for the homing of APC and trafficking of effector T cell [56]. STING deletion in DC could abrogate the capability of antigen presentation and decrease the abundance of TIL [57]. Even though cGAS-STING pathway could induce the activation of apoptosis pathway in cancer cell, the pro-apoptosis role of cGAS-STING remains to be further determined in immune cells [58, 59].

#### STING status in immune cell

Activated cGAS-STING signals could be detected in both tumor cells and immune cells. Although STING activation in tumor cells participates in anti-tumor immune response, active cGAS-STING pathway in host immune cells mainly contributes to tumor control [54]. Sivick et al. confirmed that stimulating cGAS-STING pathway in immune cells could effectively initiate anti-tumor inflammation in mouse bearing STING-deficient tumor [60]. On the contrary, by establishing mouse melanoma xenograft model (B16), Fu et al. observed that STING-deficient mice poorly responded to STING-based cancer vaccine compared with wild-type mice [61].

Among immune cells, DC acts as the core of anti-tumor immunity. The STING molecule in DC magnifies the signal from cytosolic DNA sensor and boosts tumor-specific adaptive immunity. On the one hand, DC directly takes up extracellular DNA from damaged or dying tumor cells [54, 62]. On the other hand, intracellular STING in DC could be activated by extrinsic cGAMP from tumor cell or artificially added analogues [63]. Marcus et al. investigated the influence of extracellular cGAMP in tumor microenvironment in mouse RMA-S lymphoma model [63]. Following cGAMP injection into RMA-S tumor and inhibition of Golgi transport activity, intracellular cytokine staining showed accumulating IFN-β in CD11^+^ infiltrating lymphocytes [63].

#### cGAS-STING pathway in immune regulation

In general, cGAS-STING signaling pathway is favorable to anti-cancer immune response. However, cGAS-STING was also observed as an inhibitory component in cancer immune microenvironment in some studies. In Lewis lung carcinomas, the application of STING agonist could induce immune tolerogenic state by inhibitory signal indoleamine 2,3-dioxygenase (IDO), which protects cancer cell from immune attack and promotes tumor outgrowth [64, 65]. Additionally, after intratumoral injection of high dose of STING agonist, the increased apoptosis markers and decreased cellularity were noticed in tumor-draining lymph node (TDLN) of injected side [60]. In tongue squamous cancer patients, the expression of STING was positively correlated with the abundance of regulatory T cell (Treg) [66].

As a versatile pathway, the role of cGAS-STING needs further investigation especially considering that its function changes along with agonist dose, cancer type, and disease stage [37]. Notably, advances of immunotherapy provide multiple feasible approaches to reprogram tumor immune microenvironment together with cGAS-STING agonist.

### The advances of STING agonist

#### Dimethyloxoxanthenyl acetic acid

A growing body of evidence demonstrated the important role of cGAS-STING pathway in bridging anti-tumor innate immunity and adaptive immunity [17]. Therefore, the pharmacological activation of cGAS-STING pathway would be a potential cancer treatment strategy (Table 1). Dimethyloxoxanthenyl acetic acid (DMXAA) is the first agent targeting cGAS-STING pathway [67]. Actually, DMXAA was originally designed as an anti-angiogenesis agent which was latterly found to directly interact with STING [68]. DMXAA showed potent tumor-killing effect in multiple mice models but it failed in phase III clinical trial [68]. Conlon et al. found that the interaction between DMXAA and STING was restricted in mice. In human, this interaction was too weak to induce downstream type I IFN [68].

**Table 1:** The anti-cancer effect of cGAS-STING agonist

cGAS-STING agonist	Cancer type	Agent delivery	Ref.
3′3′-cGAMP	Mouse B cell malignancies	Intraperitoneal injection	[109]
2′3′-cGAMP	Mouse lymphoma	Intratumoral injection	[63]
ML RR-S2 CDG	Mouse melanoma	Intratumoral injection	[69]
ML RR-S2 cGAMP	Mouse melanoma	Intratumoral injection	[69]
ML RR-S2 CDA (ADU-S100)	Mouse melanoma, colon cancer, mammary carcinoma	Intratumoral injection	[61, 69, 70]
DMXAA^†^	Mouse lung cancer, mesothelioma, human lung cancer, and prostate cancer	Intravenous injection	[110–112]
Cyclic di-GMP	Mouse melanoma, prostate cancer, glioma, breast cancer	Intratumoral injection	[22, 94, 113, 114]
DiABZI	Mouse colon tumor	Intravenous injection	[72]

Agent delivery listed in the table is the common delivery approach of cGAS-STING agonist. Cyclic dinucleotide encapsulated by some nanoparticles such as endosomolytic polymersomes could be delivered by intravenous injection as well [71]

*cGAMP* cyclic GMP-AMP, *CDA* cyclic di-AMP, *ML* mixed linkage, *DMXAA* 5,6-dimethylxanthenone-4-acetic acid, *DiABZI* dimeric amidobenzimidazole, *CDG* cyclic di-GMP

^†^Mouse STING-specific agonist with weak binding affinity to human STING, failing to pass phase III clinical trials

#### Cyclic dinucleotides

Motivated by effective anti-tumor effect of DMXAA in mouse model, researchers have always been trying to find an appropriate human cGAS-STING agonist. It has been verified that both host- and bacteria-derived CDN could activate cGAS-STING pathway in innate immunity. Apart from natural CDN, synthetic CDN was developed for more robust immune response [26, 28]. Artificially synthetic CDN such as mixed linkage dithio CDN (ML RR-S2 CDN) is resistant to snake venom phosphodiesterase and possesses higher binding affinity to all common human STING alleles [69]. ML RR-S2 CDA, also known as ADU-S100 developed by Aduro Biotech, has showed its anti-cancer effect in multiple mouse models [69, 70]. The in vitro experiments demonstrated that ADU-S100 could promote human peripheral blood mononuclear cell (PBMC) to generate pro-inflammation cytokines such as IFN-β [69]. In vivo experiment, Sivick et al. found that the anti-tumor effect of CDN changed along with intratumoral injection dose [60]. In mouse models, high-dose intratumoral injection of ADU-S100 (500 μg) could eliminate tumor which might largely depend on non-adaptive immunity fashions such as innate or cytotoxic mechanisms [60]. On the contrary, low-dose intratumoral ADU-S100 mainly activated adaptive anti-tumor immunity [60]. To further explore the efficacy of ADU-S100, two phase I clinical trials are ongoing.

CDNs possess the capability to induce anti-tumor inflammation in theory, but the actual treatment effect of CDNs without appropriate carrier is limited [71]. Due to the characteristics of electronegativity and high water solubility, it is hard for CDNs to cross cellular membrane and activate cytoplasmic STING [71]. Therefore, developing drug carrier with high bioavailability would be meaningful for enhancing therapeutic effect of CDNs [71]. Besides, another challenge for CDN application is drug delivery. Traditional CDN delivery by intratumoral injection has two main problems. Firstly, due to the heterogeneity among different tumor lesions even in the same individual, intratumoral injection-induced anti-tumor immunity could not cover all tumor antigen spectrum [60]. Moreover, for some inaccessible tumors, intratumoral delivery of STING agonist is an unfeasible treatment strategy [72]. Therefore, a novel delivery system or STING agonist which could be systemically delivered would be more valuable for clinical application.

#### Dimerized amidobenzimidazole

In 2018, Ramanjulu et al. reported a small molecular STING agonist with systemic anti-cancer effect [72]. This novel STING agonist was designed based on amidobenzimidazole (ABZI) which had modest binding affinity to STING subunit [72]. However, the binding affinity was significantly enhanced after dimerization by 4-carbon butane linker (di-ABZI) [72]. Human PBMC samples were collected to analyze the dose-dependent activation of STING as evaluated by IFN-β level [72]. The results showed that concentration for half maximal effect (EC50) of di-ABZI was markedly lower than cGAMP [72]. Mice bearing subcutaneous CT-26 tumor were used to assess the anti-cancer effect of di-ABZI [72]. Mice undergoing di-ABZI treatment had a great advantage in tumor control and survival data over vehicle group [72]. Notably, 80% of di-ABZI-treated mice kept tumor free until the end of the study [72].

### The application of STING agonist in immunotherapy

#### STING agonist: cancer vaccine adjuvant

Due to central and peripheral tolerance, tumor-associated antigen (TAA) is characterized by weak immunogenicity [73, 74]. Therefore, an appropriate adjuvant is essential to overcome tolerance and boost tumor-specific immunity. It is confirmed that triggering innate immunity could facilitate the activation of APC, which subsequently enhances pre-existing TAA specific or induces vigorous tumor-specific immunity [73]. In the development of cancer vaccine, multiple adjuvants are widely adopted such as live-attenuated tuberculosis vaccine [75]. When adjuvant and TAA are delivered together, Th1 skewing immune response is initiated [73]. The adjuvant function of CDN has been verified in the development of H5 influenza vaccine [76]. Given the core role of STING in the initiation of innate immunity, it was speculated that STING stimulator could serve as cancer vaccine adjuvant [27].

In multiple tumor-bearing mice models, Fu et al. firstly investigated the efficacy of STING agonist-based cancer vaccine STINGVAX which consisted of CDNs and granulocyte-macrophage colony-stimulating factor (GM-CSF)-secreting cancer cells [61]. After a single dose of STINGVAX injection into the contralateral side of the transplanted B16 melanoma, tumor growth was significantly retarded in mice, and the treatment effect was dose-dependent [61]. Compared with GM-CSF-secreting cancer cell vaccine without formulation with CDNs, tumor tissues obtained from STINGVAX-treated mice had more infiltrating CD8^+^ IFN-γ^+^ T cells [61]. In addition, the potent anti-cancer effect of STINGVAX was verified in multiple tumor-bearing mice models including colon cancer, digestive squamous cell cancer, and pancreatic cancer [61]. Further analysis showed that synthetic CDN had a strong immuno-stimulatory effect on both mouse and human DC [61]. These phenomena were later replicated in other studies, demonstrating the feasibility of using STING agonist as cancer vaccine adjuvant [77, 78].

#### STING agonist: the sensitizer of ICI treatment

ICI treatment mainly consists of anti-programmed death-1/programmed death-ligand 1 (PD-1/PD-L1) and anti-cytotoxic T-lymphocyte-associated protein 4 (CTLA-4) [79]. Anti-PD-1/PD-L1 monoclonal antibody (mAb) restores TIL from exhausted status and enhances tumor-killing activity [80, 81]. Anti-CTLA-4 mAb increases available co-stimulatory molecules (CD80 and CD86) and relieves competitive inhibition [82, 83]. Even though ICI treatment theoretically could reprogram tumor immune microenvironment and induce tumor regression, the actual clinical application is limited by low response rate.

#### Anti-PD-1/PD-L1 combined with STING agonist

STING agonist is the ideal sensitizer for anti-PD-1/PD-L1 therapy. On the one hand, STING agonist enhances ICI treatment effect. Firstly, STING agonist promotes the infiltration of T cell into tumor. Pre-existing CTL is the precondition of robust anti-PD-1/PD-L1 treatment effect. Therefore, interventions promoting T cell infiltration into tumor is helpful to relieve anti-PD-1/PD-L1 resistance. Grabosch et al. used DNA-damage-inducing agent cisplatin to activate cGAS-STING pathway in mice bearing ovarian tumor [84]. The results showed that activated cytosolic DNA-sensor enhanced T cell infiltration [84]. This transformation to “hot tumor” might relate with the expression of IFN-stimulated genes (ISG) such as *CXCL9* and *CXCL10*, which could recruit APC and T cell to tumor [85]. Secondly, cGAS-STING agonist counteracts the decrease of major histocompatibility complex (MHC) molecules on tumor cell which is an important approach to escape immune surveillance [86]. It was observed that cytosolic DNA-dependent IFN upregulation contributed to the increase of antigen presentation molecules (Tap1, Tap2, MHC I) [84]. Thirdly, cGAS-STING pathway elevates the sensitivity of tumor cell to immune killing activity of NK and CTL [50]. In the co-culture test, NK and CTL resistant tumor cell usually had higher NLRX1 and NLRC3 level, which antagonized the expression of cGAS-STING-induced type I IFN [50]. Actually, cGAS-STING pathway regulates anti-tumor immunity in a comprehensive manner from enhancing antigen presentation to increasing cytotoxicity.

On the other hand, anti-PD-1/PD-L1 therapy neutralizes the immunosuppressive effect of cGAS-STING agonist [84]. It was reported that activated cGAS-STING accompanied the upregulation of PD-L1 expression [84]. By infecting PD-L1^low^ mouse tumor cell (2F8 cell) with cGAS-STING-encoding adenovirus, nearly all infected tumor cell expressed PD-L1 while 46% of tumor cells infected by control adenovirus expressed PD-L1 [84]. Presumably, type I IFN also participates in the regulation of PD-L1 by phosphorylating JAK1-STAT1/STAT2/STAT3-IRF1 pathway, even in the less extent than IFN-γ [87].

Tan et al. formulated nanosatellite vaccine SatVax with cGAMP and antigenic peptides (Q19D, Q15L) [50]. Combination therapy of SatVax plus anti-PD-L1 significantly increased E7-specific CD8^+^ CTL but simultaneously decreased the ratio of CD8^+^ Tim3^+^ and CD8^+^ PD-1^+^ T cell in xenograft model [50]. The combination therapy induced potent tumor regression, and four of five mice achieved completely tumor-free status [50]. Similarly to the nanosatellite in SatVax, poly beta-amino ester (PBAE) nanoparticles could enhance the delivery of CDN as well [88]. In the mice bearing B16 melanoma, co-administration of PBAE-CDN and anti-PD-1 antibody markedly slowed tumor growth compared with anti-PD-1 plus unencapsulated CDN or anti-PD-1 monotherapy [88]. It was notable that mice that received STING agonist-combined anti-PD-1 treatment were resistant to tumor rechallenge in multiple xenograft models [61, 71]. We proposed that cGAS-STING-induced type I IFN might promote the survival of memory tumor-specific CTLs.

#### The role of cGAS-STING pathway in anti-CTLA-4 treatment

Anti-CTLA-4 treatment reduces the activation threshold of T cells and magnifies the tumor-specific immune response [89, 90]. Some studies revealed that anti-CTLA-4 mAb could selectively eradicate Tregs by antibody-dependent cell-mediated cytotoxicity (ADCC) effect [91, 92]. Shane et al. found intact cGAS-STING pathway was indispensable to maximized anti-CTLA-4 treatment effect [93]. Mice bearing B16 melanoma received the injection of irradiated tumor cells and subsequent anti-CTLA-4 treatment [93]. After combined treatment, no significant abscopal tumor eliminated effect was detected in mice receiving injection of STING-deficient B16 tumor cells [93]. In the meanwhile, STING deficiency markedly impaired CD8^+^ T infiltration in tumor bed [93].

Ager et al. investigated the efficacy of intratumoral injection of ICIs containing three checkpoint regulatory antibodies: anti-CTLA-4 antibody (9H10), anti-PD-1 antibody (RMP114), and agonistic anti-4-1BB antibody (3H3) in mouse prostate cancer model [94]. The results showed that the ICI cocktail therapy eliminated bilateral tumors in 40% of mice while the contaminant administration of STING agonist CDG and triple immune checkpoint blockade induced bilateral tumor regression in 75% of mice [94]. By tracking the immunodominant neoepitope SPAS expressed in the established mouse prostate tumor, it was detected that the local administration of CDG and ICIs increased SPAS-specific CD8^+^ T cell in injected tumor. Further analysis revealed that the ratio of SPAS-specific CD8^+^ T cell to total tumor-infiltrating CD8^+^ T cell decreased [94]. Therefore, CDG combined ICIs effectively expanded T cell receptor (TCR) repertoire and activated immune response targeting subdominant antigens [94].

#### The predictive role of cGAS-STING pathway in oncolytic virus

As mentioned above, intact cGAS-STING pathway is the essential competent for host to defend the invasion of DNA viruses, retroviruses, and intracellular bacterial pathogens [95–97]. In the same time, it was found that multiple cancers harbored deficient cGAS-STING pathway such as colon cancer and melanoma [46, 98]. Thus, it is logical to choose oncolytic virus strategy for cGAS-STING-deficient cancer patients [98]. Xia et al. used herpes simplex virus type 1 (HSV-1) lacking γ34.5 gene in mouse melanoma model [98]. The γ34.5 viral protein could repress host innate immunity and HSV-1Δγ34.5 could effectively activate cGAS-STING pathway and clear virus infection in normal cells [98]. However, for cGAS-STING-deficient tumor cells, disabled anti-viral response resulted in rapid virus proliferation and ultimate cell death [98]. It was displayed that melanoma cells with deficient STING were susceptible to HSV-1Δγ34.5 infection [98]. In addition, Barber et al. found that cGAS-STING-deficient mice possessed higher response rate to intratumoral injection of HSV-1Δγ34.5 in mouse ovarian cancer model [99]. Given the frequent inactivation of cGAS-STING in multiple cancers, usually caused by hypermethylation, it would be reasonable to apply oncolytic virus therapy in a subset of patients.

#### Combined STING agonist and CAR-T therapy

By transferring gene encoding CAR, engineered T cell could specifically recognize target antigen on tumor cell with single-chain variable fragment (scFv) domain [100]. CAR-modified T cell is activated independent of MHC manner and then directly kills tumor cell [101, 102]. CAR-T therapy has been successfully applied in hematological diseases, but its effect is limited in solid tumors [103, 104]. It is generally believed that immunosuppressive tumor microenvironment and intratumoral heterogeneity mainly contribute to escape from immune killing by CAR-T cell [105–107]. Smith et al. designed a novel implantable bioactive carrier which could deliver CAR-T cells to the surface of tumors [108]. Compared with systemic delivery of CAR-modified T cell, delivery by this bioactive carrier significantly enhanced T cell expansion and tumor control [108]. CAR-T therapy delivered by implanted scaffold prolonged survival time, but the intervention could not completely eliminate tumor in mice [108]. Under selective pressure, tumor cell with high expression of targeting expression (RAE1) were destroyed while RAE1^low/negative^ tumor cells survived [108]. As a result, all mice developed resistance to CAR-T therapy [108]. Then, the bioactive scaffold was modified with additional STING agonist cyclic di-GMP (cdGMP) [108]. Co-delivery of cdGMP and CAR-T cells markedly increased the activation of downstream signaling pathway of the TCR/CD3 and circulating tumor-specific T cells [108]. In mouse pancreatic tumor model, combined delivery of CAR-T cells and cdGMP completely cleared tumor in four of ten mice and significantly prolonged survival time [108]. To further investigate this combination therapy induced systemic anti-tumor immunity, four mice undergoing complete regression were re-challenged with the intravenous injection of tumor cells [108]. It was notable that the prior combination treatment inhibited the formation of measurable tumor mass [108]. We proposed that STING agonist could boost the efficacy of CAR-T-induced in situ cancer vaccine and initiate durable systemic anti-tumor immune response.

## Conclusion

Activated cGAS-STING pathway and its downstream signals boost the whole cancer-immunity cycle by enhancing cross-presentation and immune-killing activity. Therefore, cGAS-STING agonist is an ideal sensitizer for cancer immunotherapy and decreases the risk of drug resistance. On the one hand, STING agonist alters immune microenvironment from immunosuppressive type to immunosupportive type. On the other hand, as an identified adjuvant, STING agonist enhances the effect of treatment-induced in situ cancer vaccine and provides systemic memory anti-cancer effect. A growing body of evidence indicates that a cocktail of cGAS-STING agonist together with immunotherapy could effectively eradicate tumor mass and induce durable anti-tumor immune response. We believe manipulating cGAS-STING pathway might be a promising synergistic strategy with cancer immunotherapy.

## Abbreviations

ADCC: Antibody dependent cell-mediated cytotoxicity
BAX: BCL2-associated X
CAR-T: Chimeric antigen receptor T cell
cdGMP: Cyclic di-GMP
CDN: Cyclic-dinucleotide
cGAMP: Cyclic GMP-AMP
cGAS: Cyclic GMP-AMP synthase
CTLA-4: Cytotoxic T-lymphocyte-associated protein 4
CXCL9: Chemokine (C-X-C motif) ligand 9
DC: Dendritic cell
DMXAA: Dimethyloxoxanthenyl acetic acid
dsDNA: Double-stranded DNA
EC50: Concentration for half maximal effect
ER: Endoplasmic reticulum
GM-CSF: Granulocyte-macrophage colony-stimulating factor
HSV-1: Herpes simplex virus type 1
ICI: Immune checkpoint inhibitor
IDO: Indoleamine 2,3-dioxygenase
IFN: Interferon
IKK: IκB kinase
IRF3: Interferon regulatory transcription factor 3
ISG: IFN-stimulated genes
mAb: Monoclonal antibody
ML: Mixed linkage
NF-κB: Nuclear factor kappa-light-chain-enhancer of activated B cell
NIK: NF-κB-inducing kinase
PBAE: Poly beta-amino ester
PD-1: Programmed death-1
PD-L1: Programmed death-ligand 1
PRR: Pattern recognition receptor
ROS: Reactive oxygen species
scFv: Single-chain variable fragment
STING: Stimulator of interferon genes
TAA: Tumor-associated antigen
TBK1: TANK-binding kinase 1
TCR: T cell receptor
TDLN: Tumor-draining lymph node
Treg: Regulatory T cell
